# Effect of AlF_3_ on the Density and Elastic Properties of Zinc Tellurite Glass Systems

**DOI:** 10.3390/ma5081361

**Published:** 2012-08-13

**Authors:** Haji Abdul Aziz Sidek, Shaharuddin Rosmawati, Mohamed Kamari Halimah, Khamirul Amin Matori, Zainal Abidin Talib

**Affiliations:** Glass Ceramic Composite Research Group (GCCG), Department of Physics, Faculty of Science, Universiti Putra Malaysia, 43400 UPM Serdang, Selangor, Malaysia; E-Mails: rosma_shaha@yahoo.com (S.R.); halimah@science.upm.edu.my (M.K.H.); khamirul@science.upm.edu.my (K.A.M.); zainalat@science.upm.edu.my (Z.A.T.)

**Keywords:** tellurite glass, zinc oxide, physical properties, elastic moduli

## Abstract

This paper presents the results of the physical and elastic properties of the ternary zinc oxyfluoro tellurite glass system. Systematic series of glasses (AlF_3_)_x_(ZnO)_y_(TeO_2_)_z_ with x = 0–19, y = 0–20 and z = 80, 85, 90 mol% were synthesized by the conventional rapid melt quenching technique. The composition dependence of the physical, mainly density and molar volume, and elastic properties is discussed in term of the AlF_3_ modifiers addition that are expected to produce quite substantial changes in their physical properties. The absence of any crystalline peaks in the X-ray diffraction (XRD) patterns of the present glass samples indicates the amorphous nature. The addition of AlF_3_ lowered the values of the densities in ternary oxyfluorotellurite glass systems. The longitudinal and transverse ultrasonic waves propagated in each glass sample were measured using a MBS8020 ultrasonic data acquisition system. All the velocity data were taken at 5 MHz frequency and room temperature. The longitudinal modulus (L), shear modulus (G), Young’s modulus (E), bulk modulus (K) and Poisson’s ratio (σ) are obtained from both velocities data and their respective density. Experimental data shows the density and elastic moduli of each AlF_3_-ZnO-TeO_2_ series are found strongly depend upon the glass composition. The addition of AlF_3_ modifiers into the zinc tellurite causes substantial changes in their density, molar volume as well as their elastic properties.

## 1. Introduction

Tellurite glasses are a relatively new vitreous material and appropriate candidates for new optical materials due to their excellent properties, such as high refractive index, high dielectric constants, a wide band infrared transmittance and substantial third order non-linear optical susceptibility. Their low melting temperatures and non-hygroscopic nature, which limit applications of phosphate and borate glasses, make them of much current interest [1,2]. It has been established that the basic structure of these tellurite glasses is a TeO_4_ trigonal bipyramid (tbp) with a lone pair of electrons in one of its equatorial sites [2]. 

The binary zinc oxide-tellurite glasses have been systematically studied where their densities increase with substitution of up to 40 mol% ZnO into the TeO_2_ network system. However, their molar volume decreases [3]. The addition of ZnO into the binary tellurite glass network reveals opposite behaviors to that of ZnCl_2_ [4], which are closely related to the structural changes.

The linear increase of the elastic modulus of ZnCl_2_-TeO_2_ glasses is associated with the force constants (f) for a constant mean ring size of each of the atoms involved [2]. The elastic moduli of ZnCl_2_-TeO_2_ glasses also increase due to cross-links for force constants of vitreous tellurite networks. The change in the force constant of 10% can be obtained by altering one Te-O bond into a Te-Cl bond and one Zn-Cl bond into a ZnO bond. This is most likely the cause of the higher modulus and hence a reduction in the size of rings. However there are no coordination number changes in substituting Cl atoms with O atoms in ZnCl_2_-TeO_2_ glasses. 

The sound velocities at a frequency of 10 MHz and the elastic moduli for a Na_2_O-ZnO-B_2_O_3_ glass system as a function of ZnO concentration have been studied recently [5]. Both sound velocities and elastic moduli were found to increase with the addition of ZnO content. Poisson’s ratio and Debye temperature were also found to increase with ZnO concentration. The results indicate that the Zn^2+^ ions are likely to occupy network forming positions in this glass system. 

Meanwhile, both longitudinal and shear ultrasonic velocities, as well as their elastic moduli decreased with increasing ZnF_2_ mol% content in the TeO_2_-WO_3_-ZnF_2_ glasses [6]. In this case, the addition of ZnF_2_ to the tellurite glass matrix weakens the structure by opening the network where the formation of non-bridging oxygen (NBO) is most influenced by the halide ions effect. 

The objective the current work is to study the effect of AlF_3_ on the density and the elastic properties of zinc tellurite glass systems. The short-term significance of this work is to establish a baseline for the elastic properties of vitreous zinc tellurite with the addition of AlF_3_ into the glass network.

## 2. Results and Discussion

**Figure 1:**
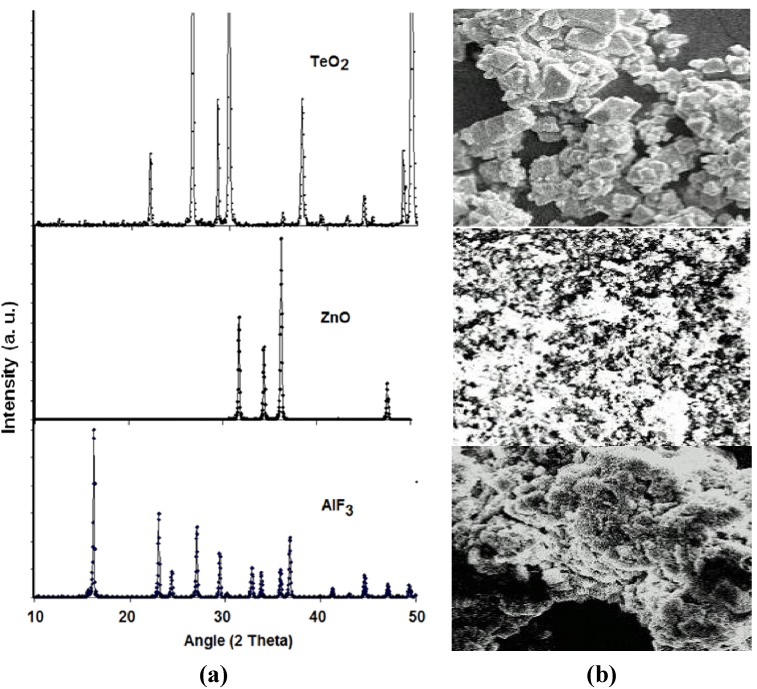
(**a**) The X-ray diffraction (XRD) pattern; and (**b**) the corresponding scanning electron microscope (SEM) photographs of chemical powders of TeO_2_ (Technical Grade), ZnO (99.9%) and AlF_3_ (97.0%).

**Figure 2:**
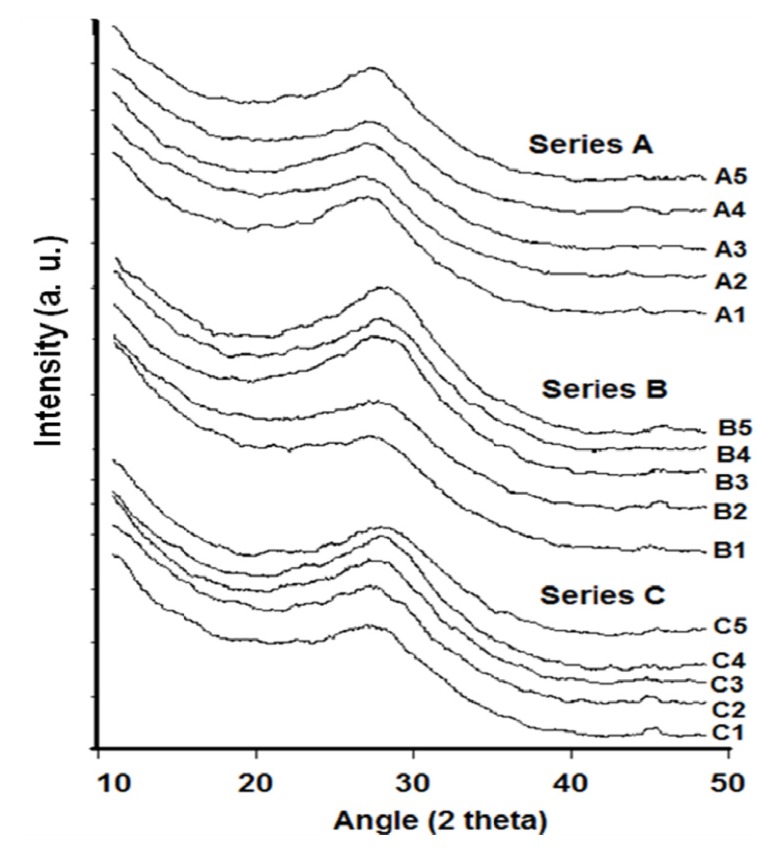
The XRD patterns of ternary zinc oxyfluoro tellurite glass series.

Figure 1 shows the X-ray diffraction (XRD) patterns of (the chemical compositions) and the corresponding scanning electron microscope (SEM) photographs of crystalline tellurite (TeO2), zinc oxide (ZnO) and aluminum fluoride (AlF3) while the XRD patterns of zinc oxyfluoro tellurite glass samples obtained are shown in Figure 2. As depicted in Figure 2, all the zinc oxyfluorotellurite glass series were found to show no discrete or continuous sharp peaks but a broad halo at around 2Θ ≅ 26°–30°, which reflected the characteristic amorphous glass structure. This indicates the absence of a long-range atomic arrangement and the periodicity of the three dimensional network in the quenched material [7,8]. 

**Figure 3:**
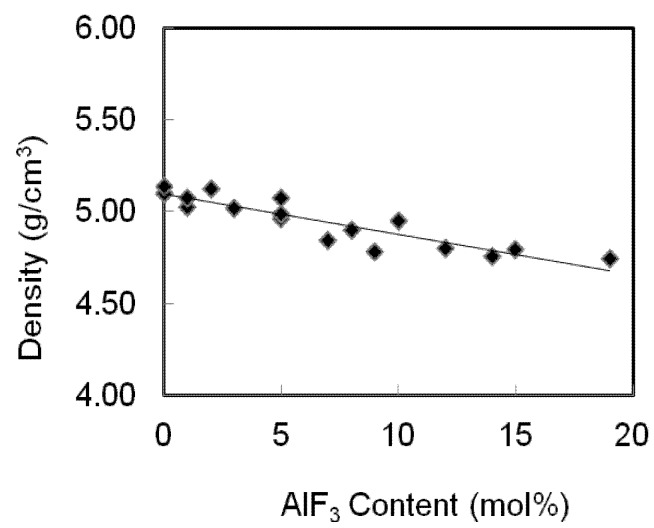
Variation of density of AlF_3_-ZnO-TeO_2_ glasses. The line is drawn to guide the eye.

The densities and molar volumes for an AlF_3_ zinc tellurite glass system are presented in Table 1 and shown in Figure 3. The densities of TeO_2_-ZnO-AlF_3_ samples decrease as the AlF_3_ was added to substitute the ZnO content. These results are in reasonable agreement with the statement of Mallawany’s that the halogen substitution lowered the density [9]. Even the addition of ZnCl into the oxychloride glass system (TeO_2_-ZnO-ZnCl) also lowered the densities of the TeO_2_-ZnO glass system [10].

**Table 1:** Glass composition, density, molar volume, molecular weight, longitudinal and shear ultrasonic wave velocities of (AlF_3_)_x_-(ZnO)_y_-(TeO_2_)_z_ glasses. The pure TeO_2_ glass is included for comparison.

Glass sample	Composition (mol%)			Density (kg·m^−3^)	Molar volume (cm^3^/mol)	Molar weight (g/mol)	V_l_	Vs
	AlF_3 x_	ZnO_y_	TeO_2z_				(m s^−1^)	
Pure	0	0	100	4806	33.21	159.61	3435	
A1	0	10	90	5098	29.77	151.77	3324	
A2	1	9	90	5023	30.22	151.80	3316	
A3	3	7	90	5018	30.26	151.84	3364	
A4	5	5	90	4963	30.61	151.92	3393	
A5	7	3	90	4846	31.36	151.97	3424	
A6	9	1	90	4779	31.81	152.02	3435	
B1	0	15	85	5102	28.98	147.86	3307	
B2	1	14	85	5075	29.14	147.89	3334	
B3	5	10	85	4990	29.66	148.00	3409	
B4	8	7	85	4898	30.23	148.07	3480	
B5	12	3	85	4799	30.88	148.19	3486	
B6	14	1	85	4756	31.65	150.53	3488	
C1	0	20	80	5136	28.03	143.96	3296	
C2	2	18	80	5124	28.1	143.98	3353	
C3	5	15	80	5074	28.4	144.10	3398	
C4	10	10	80	4950	28.33	140.23	3471	
C5	15	5	80	4792	28.4	136.09	3528	
C6	19	1	80	4743	30.45	144.42	3542	

The densities decrease gradually with the addition of AlF_3_ content. In this case, as more AlF_3_ content are added into the zinc tellurite glass system, two Zn-F single bonds will replace the Zn=O double bonds, hence the structure of the glasses becomes loose, which results in a decrease in density. Fluorine plays an important role in the glass matrix; replacing the O^2−^ ions causes the compositional dependence of the density and molar volume [11]. The molecular weight of TeO_2_ is 159.599 which is greater than AlF_3_ (83.977) and ZnO (81.408). Whereas the atomic weight of individual constituent atom Te (127.6) > Zn (65.39) > Al (26.9815) > F (18.998) > O (15.9999), the atomic radius of each of the constituent atoms (in ppm) is Al (143) > Te (140) > Zn (134) > O (66) > F (64).

The slight increase in molar volumes is due to the rearrangement of the lattice and a decrease in the porosity of the glass. The increase in molar volumes for all series in ternary glass systems as shown Table 1 is related to a decrease in the bond length or inter-atomic spacing between the atoms. The radius of Te^2+^ (0.097 nm) is much greater than that of Zn^2+^ (0.074 nm) and since the radius of Al^3+^ is 0.0535 nm which also has a smaller radius than Zn^2+^ attributed to this situation. 

The molar volumes for AlF3-ZnO-TeO2 results show slightly lower values than that of the pure tellurite glasses. The much higher reduction in the molar volume for the sample containing fluorine is attributed to the decrease in viscosity due to the breaking of the Te-O-Te bond to form two Te-F bonds, which increase the efficiency of the crystallization process [12]. 

The addition of AlF_3_ that consists of Al^3+^ and F will modify the glass structure by creating NBOs in the oxyflorotellurite glass system. The NBOs created were believed to alter the glass structure in a way that packing of the molecule becomes denser as more network modifier ions (in this case Al^3+^), attempt to occupy the interstices within the network. 

**Figure 4:**
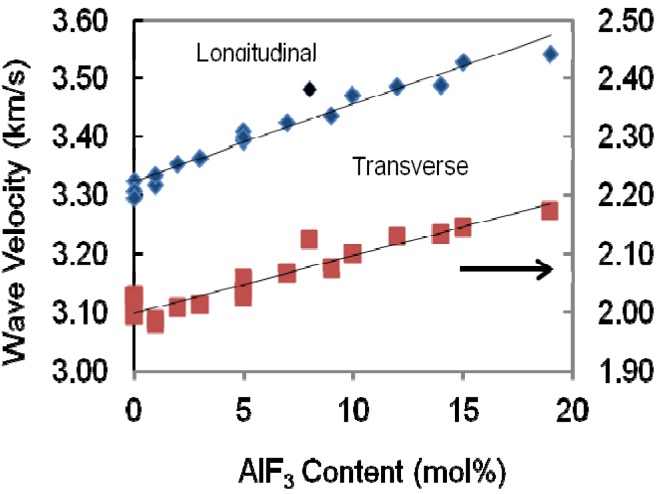
Variation of longitudinal and transverse wave velocities propagated in the AlF_3_-ZnO-TeO_2_ glasses.

The variation of both longitudinal and shear wave velocities that propagated in the present bulk samples depends on the structural change of the glass network. The longitudinal and shear ultrasonic velocities in ternary glass TeO_2_-ZnO-AlF_3_ are depicted in Figure 4 and presented in Table 1 for different mole fractions of AlF_3_ content. It can be seen that in the glasses studied, both longitudinal and shear ultrasonic velocities increase when more AlF_3_ is added into the ZnO-TeO_2_ glass system.

**Figure 5:**
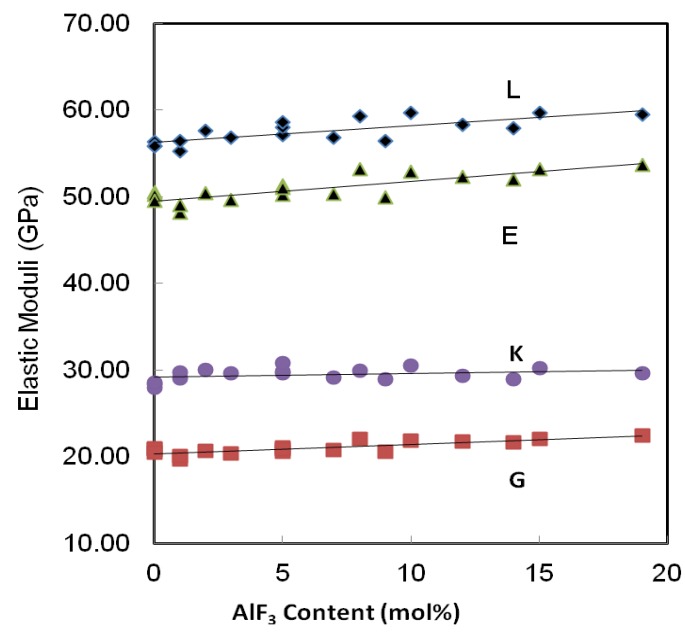
Variation of elastic moduli in the AlF_3_-ZnO-TeO_2_ glasses. The line is drawn to guide the eye.

The percentage increase of velocity can be seen in Table 2. The percentage of decrement in velocity increases with the increasing substitution of AlF_3_ content. Substituting more AlF_3_ content to ZnO content means that the difference between maximum velocity and minimum velocity is higher for both longitudinal and transverse or shear velocity. The addition of AlF_3_ content increase both wave velocities propagated in glass, and hence the elastic stiffness is also increased [13] as shown in Figure 5.

**Table 2:** The elastic moduli, Young’s modulus (E), longitudinal modulus (L), shear modulus (G), bulk modulus (K) and Poisson’s ratio (σ) of ternary zinc tellurite glass.

Glass sample	Elastic moduli (GPa)					σ	d = 4G/K
	L	G	E	K	H		
Pure	56.71	21.50	51.37	28.04	4.38	0.19	3.07
A1	56.33	21.01	50.53	28.32	4.17	0.20	2.97
A2	55.23	19.67	48.13	29.00	3.63	0.22	2.71
A3	56.79	20.33	49.66	29.67	3.78	0.22	2.74
A4	57.14	20.61	50.21	29.65	3.88	0.22	2.78
A5	56.81	20.72	50.27	29.18	3.97	0.21	2.84
A6	56.39	20.58	49.91	28.95	3.94	0.21	2.84
B1	55.80	20.90	50.18	27.93	4.17	0.20	2.99
B2	56.41	20.04	49.07	29.70	3.68	0.22	2.70
B3	57.99	21.11	51.25	29.84	4.03	0.21	2.83
B4	59.32	22.08	53.14	29.88	4.36	0.20	2.96
B5	58.32	21.75	52.32	29.32	4.31	0.20	2.97
B6	57.86	21.66	52.02	28.98	4.32	0.20	2.99
C1	55.80	20.44	49.51	28.54	3.94	0.21	2.86
C2	57.61	20.68	50.46	30.03	3.86	0.22	2.75
C3	58.59	20.83	50.99	30.82	3.83	0.22	2.70
C4	59.64	21.83	52.88	30.53	4.20	0.21	2.86
C5	59.64	22.05	53.21	30.25	4.31	0.21	2.92
C6	59.50	22.42	53.70	29.62	4.52	0.20	3.03

As a small quantity of AlF_3_ is added into the TeO_2_-ZnO glass network, a breaking of Te-O-Te takes place. The conversion of this linkage results in depolymerization of the network leading to the formation of Te-_ax_O_eq_-Te bridges with the appearance of non-bridging oxygen (NBO) [14]. Therefore, the continuous breaking of Te-O-Te linkages with the addition of AlF_3_ content leads to the loose packing of the glass network and hence, a decrease in density (Figure 3). The observed results can be further substantiated by revealing the composition dependent on ultrasonic parameters. 

It is inferred that a decrease in Te coordination number (N) has resulted with the increase in the modifier content [15]. Further, a decrease in the coordination number results in a slight decrease in the mean Te-O bond length (R) [16]. Thus, it is inferred that the observed continuous increase in sound velocity in the present glasses is due to the change in coordination number with the substitution of AlF_3_. 

The introduction of fluorine into TeO_2_-based glass system results in a reduction of Te-O-Te linkages due to a gradual transformation of trigonal bipyramid TeO_4_ (tbp) through TeO_3+1_ to trigonal pyramid TeO_3_, decreasing the connectivity of the tellurite glass former network [17]. This behavior is strengthened by the higher concentration of F^−^ ions.

Elastic properties (longitudinal, transverse/shear, bulk and Young’s moduli, Debye temperature and Poisson’s ratio) of the present glasses have been determined from the measured ultrasonic velocities and densities. Table 2 gives experimental values of the elastic moduli: Young’s modulus (E), longitudinal modulus (L), shear modulus (G), bulk modulus (K) and Poisson’s ratio (σ). 

Similarly, all the elastic moduli show a monotonic increase as that of velocities, with an incremental increase of AlF_3_ into the ZnO-TeO_2_ glass system for every series. For series A, the Young’s modulus increases from 48.13 to 49.91 GPa. This pattern is applied to the rest of the glass series. The additional increase of AlF_3_ results in higher network rigidity, which in turns results in an increase of the longitudinal and shear modulus. The increase in shear modulus, G, and bulk modulus, K, is due to the stronger tendency of the change in the coordination number with increasing AlF_3_ content. 

In ternary glasses both the degree of cross-linking and the relative proportions of different types of bonds may be changed with composition. The existence of AlF_3_ and the nature of TeO_2_ not only cause an increase in the elastic moduli, but also an increase in Poisson’s ratio. 

**Figure 6:**
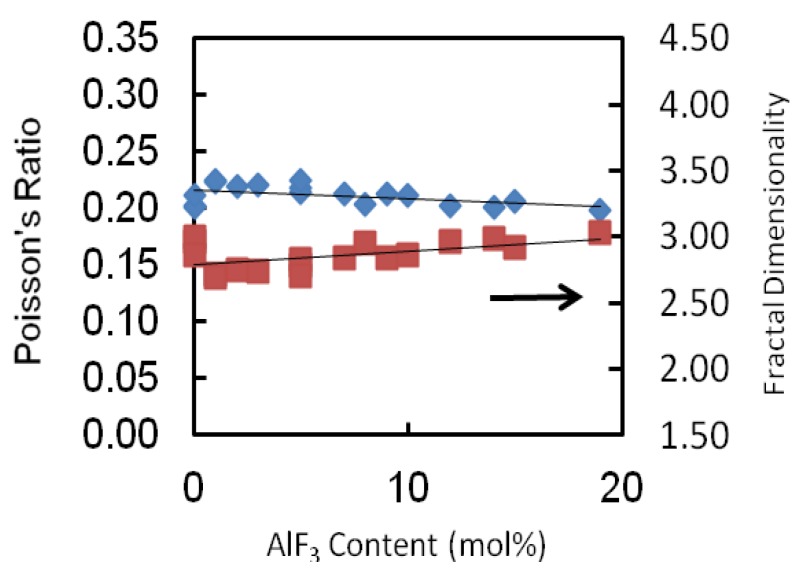
Poisson ratio and fractal dimensionality of AlF_3_-ZnO-TeO_2_ glasses. The line is drawn to guide the eye.

The values of Poisson’s ratio of each glass series is tabulated in Table 2 and depicted in Figure 6. The Poisson’s ratio shows a monotonic decrease as the AlF_3_ content increases for all glass series. For this glass series as an example, the Poisson’s ratio increases from 0.223 to 0.213. The values of the Poisson’s ratio are of the order 0.2 which reveals a high cross-link density [18,19]. For all series of glass, the transformation of cross-linkage is negligibly small (changes of about 0.02), almost remaining constant.

Table 2 collects the calculated values of microhardness. It can be seen that the microhardness of the studied glass samples, except for Series A, increases with the addition of AlF_3_ content. As an example, for Series B, the microhardness increases from 4.17 to 4.32 GPa. The rest of glass samples show the same trend. It also indicates a strengthening in the glass structure which may be due to the introduction of stronger ionic bonds in the glass network [19,20].

The shear to bulk modulus ratio of oxyfluorotellurite glasses at room temperature is given in Table 2. The *G*/*K* tends to decrease with increasing AlF_3_ content: consistent with the loss or weakening of cross-link between TeO_2_ chains. In this study, the fractal dimensionality, (*d*) of these glasses ranges between 2.70 and 3.03. These suggest an intermediate connectivity in a three-dimensional disordered network, which implies a marked degree of cross-linkage between TeO_2_ chains, as would be anticipated for modifier cations whose valency is greater than unity, or increased branching of the network of TeO_2_ chains facilitated by an increase in the number of the end and branching units incorporated into the basic tellurite network.

All the current experimental data were analyzed using Microsoft Excel, by fitting regression curves, with the results of the regression coefficients presented in Table 3. The regression coefficients obtained from each curve is shown in Figure 3, Figure 4, Figure 5 and Figure 6. In Table 3, $\hat{Y}$ stands for the variables shown in the first column and $\hat{x}$ is the ZnO concentration. As can be seen in previous figures, for most of the variables, a linear curve ($\hat{Y}$ = β$\hat{x}$ + α) gives the best fit. Except for bulk modulus, Poisson’s ratio and fractal dimensionality, d, the R^2^ values lie between 0.527 to 0.9384. 

**Table 3:** Linear regression analysis of the variables ($\hat{Y}$ stands for the variables shown in the first column and $\hat{x}$ is the ZnO concentration. As can be seen in previous figures, for most of the variables, a linear curve ($\hat{Y}$ = β$\hat{x}$ + α) for various properties of glass (density; ρ longitudinal and shear ultrasonic velocities, *v_l_*, *v_s_*; elastic moduli, *L*, *G*, *K*, *E*; Poisson’s ratio, σ; and fractal dimensionality, d).

Variables ($\hat{Y}$)	β	α	R^2^	% change
Density, ρ	−0.022	5.095	0.832	−8.286
Longitudinal Wave Velocity, V_L_	0.013	3.323	0.938	+7.464
Transverse Wave, V_S_	0.010	1.998	0.894	+9.853
Longitudinal Modulus, L	0.189	56.294	0.578	+7.984
Shear Modulus, G	0.1079	20.348	0.651	+13.98
Young’s Modulus, E	0.225	49.52	0.668	+11.573
Bulk Modulus, K	0.046	29.163	0.120	+10.347
Poisson’s Ratio, σ	−0.001	0.217	0.275	−9.998
Fractal Dimensionality, d	0.01	2.794	0.275	+12.222

The overall results from Table 3 show that the addition of AlF_3_ less than 19 mol% into the zinc tellurite glass system causes a small effect (less than 12%) on their physical properties as well as their elastic properties. However, it is difficult to completely separate the effects of increasing the AlF_3_ content and decreasing the ZnO content from the observed changes in glass properties.

## 3. Experimental Procedure

All glass samples were prepared by rapid melting quenching method. The ternary system consists of (TeO_2_)_x_ (ZnO)_y_ (AlF_3_)_z_ with x, y and z being the mole fractions. The aluminum fluoride, AlF_3_, and zinc oxide, ZnO, act as a glass modifier, while tellurite, TeO_2 _is a glass former. Table 1 shows the initial composition (mol%): density, molar volume, molecular weight, longitudinal and shear ultrasonic wave velocities of (AlF3)_x_-(ZnO)_y_-(TeO2)_z_ glasses. 

All the glass samples were prepared from commercial powders by mixing the specific weights of batches using tellurium (IV) oxide, TeO_2_ (Technical grade, Alfa Aesar, Ward Hill, MA, USA), zinc oxide, ZnO (99.99%, Assay, Alfa Aesar, Ward Hill, MA, USA) and aluminum fluoride, AlF_3_ (97%, Assay, Alfa Aesar, Ward Hill, MA, USA). Those starting materials were weighed using an electronic balance to obtain 20 g batches and mixed in the alumina crucible. Their initial glass compositions are presented in Table 1.

For the premelting process, a lidded alumina crucible which contained well mixed 20 g batches were then preheated in a preliminary furnace to 400 °C for 30 minutes. This process was carried out to evaporate water vapor and remove gases in the mixture and to allow the tellurite to decompose and react with other batch constituents before the melting process occurs [21]. The mixture of starting materials was then transferred into a second electric furnace and kept at 750 °C–800 °C for 1 hour. During the process, the crucible was slightly shaken using several times using a metal holder to ensure homogeneity and proper mixing. All the melting processes were performed using an electric furnace manufactured by Lindberg, Thermolyne and Bole.

After the melting process, each melt was rapidly quenched into a cylindrical stainless steel split mould which had been preheated to 400 °C for the glass casting process. The samples formed a glass rod of 2.0 cm height and 1.1 cm diameter. The reason to maintain the mould at this temperature is to relieve the mechanical stress in the glass sample. Then, the mould halves were released to prevent cracking [19]. Each glass sample was then annealed at 350 °C for about an hour before the furnace was switched off. The glass samples were allowed to cool down *in situ* to room temperature for a day.

All the ternary glasses were free of bubbles, of exceptional quality and yellow in color. These samples were then cut into the required dimension using an Isomet Low Speed Saw machine (Buehler). The thickness of the glass sample was taken five times for each measurement. The glass samples were then ground using various grades of sand paper. The grades used were 150, 300, 600, 1000, 1200, 2000 and 2700. This was done to obtain parallel, smooth and clear surfaces for each glass sample. There were three series of zinc oxyfluoride tellurite glass system, namely A, B and C, where each series consisted of six samples as shown in Table 1.

The densities (ρ) of the glasses were determined by the Archimedes method with acetone as buoyant liquid [22]. All the glass samples’ weights were measured with a digital balance (±0.0001 g accuracy). Their molar volume was calculated from the molecular weight (*M*) and density (ρ). The accuracy in the measurement of the density is ±0.01 g·cm^−3^ and the relative error is ±0.05%.

The chemically estimated elemental composition values present in the glass samples with the Atomic Absorption Studies (Perkin-Elmer, Model 1372, Waltham, MA, USA) were found to be slightly smaller than the corresponding elemental nominal composition values (before the glass formation), which we considered to be due to evaporation losses and uncertainties in the chemical analysis.

All the glass samples were checked by X-ray diffraction for their amorphous nature using an X’Pert Pro Panalytical PW 3040 MPD X-ray powder diffractometer by employing Cr-K*a* radiation. 

For the measurements of ultrasonic velocity in each glass sample, the samples were shaped into a circular disc of 12 mm diameter and 10–12 mm thickness. The opposite faces of the disc shaped glass samples were highly polished using ultra fine lapping papers to achieve a good surface finish with plane parallelism having an accuracy of ±5 micron.

Ultrasonic velocity measurements were carried out at a frequency of 10 MHz using x-cut and y-cut quartz transducers. A pulse superposition technique was employed using Ultrasonic Data Acquisition System (MATEC 8020, Matec Instruments, Northborough, MA, USA) [23]. Burnt honey was used as a bonding material between the glass samples and transducers. By measuring the thickness of the sample (*d*), longitudinal (*V*_l_) and transverse (*V*_t_) wave velocities were calculated using the relationship, *V* = 2*d*/*t* [24]. The absolute accuracy in the measurement of the velocity is ±5 ms^−1^ and the relative error is ±0.1%. Glasses are isotropic and have only two independent elastic constants, *L* and *G*, which can be obtained from their longitudinal and shear sound wave velocities and densities. The various elastic properties of the glasses were calculated using the following standard relations [25]:

Longitudinal modulus* L* = ρ*V*_1_^2^ (1)

Shear modulus *G* = ρ*V*_s_^2^ (2)

(3) $$\text{Bulk modulus }K=\rho \left({V_{l}}^{2}-\frac{4}{3}{V_{s}}^{2}\right)$$

(4) $$\text{Young’s modulus }E=\frac{\rho {V_{s}}^{2}\left(3{V_{l}}^{2}-4{V_{s}}^{2}\right)}{{V_{l}}^{2}-{V_{s}}^{2}}$$

(5) $$\text{Poisson’s ratio }\sigma =\frac{\left({V_{l}}^{2}-2{V_{s}}^{2}\right)}{2\left({V_{l}}^{2}-{V_{s}}^{2}\right)}$$

Fractal dimensionality* d = 4G/K* (6)

## 4. Conclusions

As a conclusion, a systematic series of zinc oxyfluoro tellurite glass systems have been successfully prepared and characterized in order to obtain their physical properties. Based on the results it showed that their densities decrease when glass modifier AlF_3_ is added into the zinc tellurite glass systems, while molar volumes increases. The decrease in density is also probably caused by a change in crosslink density and coordination number of Te^2+^ ions. The increase in the molar volume may be attributed to an increase in the bond length or interatomic spacing between the atoms. Ultrasonic properties such as velocity and elastic constant were affected considerably by glass composition. For the ternary oxyfluorotellurite glass system, it is obvious that the values of both velocities and other elastic moduli increase as more AlF_3 _is added into the zinc tellurite-based glass system. The observed continuous increase in sound velocity in the present glasses is due to the change in coordination number with the substitution of AlF_3_.

## Supplementary Materials

Supplementary File 1
