# Correction: Effect of AlF_3_ on the Density and Elastic Properties of Zinc Tellurite Glass Systems. *Materials* 2012, *5*, 1361-1372.

**DOI:** 10.3390/ma5122872

**Published:** 2012-12-13

**Authors:** Haji Abdul Aziz Sidek, Shaharuddin Rosmawati, Mohamed Kamari Halimah, Khamirul Amin Matori, Zainal Abidin Talib

**Affiliations:** Glass Ceramic Composite Research Group (GCCG), Department of Physics, Faculty of Science, Universiti Putra Malaysia, 43400 UPM Serdang, Selangor, Malaysia; E-Mails: rosma_shaha@yahoo.com (S.R.); halimah@science.upm.edu.my (M.K.H.); khamirul@science.upm.edu.my (K.A.M.); zainalat@science.upm.edu.my (Z.A.T.)

Due to an oversight, the data of the last column “Vs” in Table 1 was missing in the original version of this article [1].

**Table 1:** Glass composition, density, molar volume, molecular weight, longitudinal and shear ultrasonic wave velocities of (AlF_3_)_x_-(ZnO)_y_-(TeO_2_)_z_ glasses. The pure TeO_2_ glass is included for comparison.

Glass sample	Composition (mol%)			Density (kg·m^−3^)	Molar volume (cm^3^/mol)	Molar weight (g/mol)	V_l_	Vs
	AlF_3 x_	ZnO_y_	TeO_2z_				(m s^−1^)	
Pure	0	0	100	4806	33.21	159.61	3435	2115
A1	0	10	90	5098	29.77	151.77	3324	2030
A2	1	9	90	5023	30.22	151.80	3316	1979
A3	3	7	90	5018	30.26	151.84	3364	2013
A4	5	5	90	4963	30.61	151.92	3393	2038
A5	7	3	90	4846	31.36	151.97	3424	2068
A6	9	1	90	4779	31.81	152.02	3435	2075
B1	0	15	85	5102	28.98	147.86	3307	2024
B2	1	14	85	5075	29.14	147.89	3334	1987
B3	5	10	85	4990	29.66	148.00	3409	2057
B4	8	7	85	4898	30.23	148.07	3480	2123
B5	12	3	85	4799	30.88	148.19	3486	2129
B6	14	1	85	4756	31.65	150.53	3488	2134
C1	0	20	80	5136	28.03	143.96	3296	1995
C2	2	18	80	5124	28.1	143.98	3353	2009
C3	5	15	80	5074	28.4	144.10	3398	2026
C4	10	10	80	4950	28.33	140.23	3471	2100
C5	15	5	80	4792	28.4	136.09	3528	2145
C6	19	1	80	4743	30.45	144.42	3542	2174

We apologize for any inconvenience this may have caused.
