# Supplementary material for: Effect of AlF3 on the Density and Elastic Properties of Zinc Tellurite Glass Systems
Source: Materials (Basel). 2012 Aug 13;5(8):1361–72. doi: 10.3390/ma5081361 (PMC5448930; doi:10.3390/ma5081361)
Supplement: Supplementary File 1 [file materials-05-01361-s001.pdf]

Materials **2012**, *5*, 2872-2873; doi:10.3390/ma5122872

OPEN ACCESS

materials

ISSN 1996-1944

www.mdpi.com/journal/materials

Correction

Correction: Effect of AlF_3 on the Density and Elastic Properties of Zinc Tellurite Glass Systems. *Materials* 2012, 5, 1361-1372.

Haji Abdul Aziz Sidek *, Shaharuddin Rosmawati, Mohamed Kamari Halimah, Khamirul Amin Matori and Zainal Abidin Talib

Glass Ceramic Composite Research Group (GCCG), Department of Physics, Faculty of Science, Universiti Putra Malaysia, 43400 UPM Serdang, Selangor, Malaysia; E-Mails: rosma_shaha@yahoo.com (S.R.); halimah@science.upm.edu.my (M.K.H.); khamirul@science.upm.edu.my (K.A.M.); zainalat@science.upm.edu.my (Z.A.T.)

* Author to whom correspondence should be addressed; E-Mail: sidek@science.upm.edu.my; Tel.: +603-8946-6646; Fax: +603-8943-2508.

Received: 13 December 2012 / Accepted: 13 December 2012 / Published: 13 December 2012

Due to an oversight, the data of the last column "Vs" in Table 1 was missing in the original version of this article [1].

Table 1. Glass composition, density, molar volume, molecular weight, longitudinal and shear ultrasonic wave velocities of $(AlF_3)_x$-$(ZnO)_y$-$(TeO_2)_z$ glasses. The pure TeO_2 glass is included for comparison.

Glass sample	Composition (mol%)			Density ($kg{\cdot}m^{-3}$)	Molar volume (cm^3/mol)	Molar weight (g/mol)	V_l	Vs
	$AlF_{3\,x}$	ZnO_y	TeO_{2z}				($m\ s^{-1}$)	
Pure	0	0	100	4806	33.21	159.61	3435	2115
A1	0	10	90	5098	29.77	151.77	3324	2030
A2	1	9	90	5023	30.22	151.80	3316	1979
A3	3	7	90	5018	30.26	151.84	3364	2013
A4	5	5	90	4963	30.61	151.92	3393	2038
A5	7	3	90	4846	31.36	151.97	3424	2068
A6	9	1	90	4779	31.81	152.02	3435	2075
B1	0	15	85	5102	28.98	147.86	3307	2024
B2	1	14	85	5075	29.14	147.89	3334	1987
B3	5	10	85	4990	29.66	148.00	3409	2057
B4	8	7	85	4898	30.23	148.07	3480	2123

Table 1. *Cont.*

B5	12	3	85	4799	30.88	148.19	3486	2129
B6	14	1	85	4756	31.65	150.53	3488	2134
C1	0	20	80	5136	28.03	143.96	3296	1995
C2	2	18	80	5124	28.1	143.98	3353	2009
C3	5	15	80	5074	28.4	144.10	3398	2026
C4	10	10	80	4950	28.33	140.23	3471	2100
C5	15	5	80	4792	28.4	136.09	3528	2145
C6	19	1	80	4743	30.45	144.42	3542	2174

We apologize for any inconvenience this may have caused.

References

1. Sidek, H.A.A.; Rosmawati, S.; Halimah, M.K.; Matori, K.A.; Talib, Z.A. Effect of AlF_3 on the Density and Elastic Properties of Zinc Tellurite Glass Systems. *Materials* **2012**, *5*, 1361–1372.

© 2012 by the authors; licensee MDPI, Basel, Switzerland. This article is an open access article distributed under the terms and conditions of the Creative Commons Attribution license (http://creativecommons.org/licenses/by/3.0/).
